# ESBL *Escherichia coli* Ventriculitis after Aneurysm Clipping: A Rare and Difficult Therapeutic Challenge

**DOI:** 10.1155/2015/694807

**Published:** 2015-04-29

**Authors:** F. A. Zeiler, J. Silvaggio

**Affiliations:** Section of Neurosurgery, University of Manitoba, Winnipeg, MB, Canada R3A 1R9

## Abstract

*Background*. Extended spectrum beta-lactamase (ESBL) produced *Escherichia coli* (*E. coli*) ventriculitis is a rare infection of the central nervous system, with increasing rarity in the adult population. The therapeutic strategy to achieve cure may need to involve a combination of intraventricular and intravenous (IV) therapy. *Objective*. To describe a case of ESBL *E. coli* meningitis/ventriculitis in an adult and outline the antimicrobial therapy that leads to cure. *Methods*. We retrospectively reviewed the records of a patient admitted to the neurosurgical department for aneurysmal subarachnoid hemorrhage, who developed ESBL *E. coli* ventriculitis. *Results*. A 55-year-old female, admitted for a Fisher grade 3, World Federation of Neurological Surgeons grade 1, subarachnoid hemorrhage, developed ESBL *E. coli* ventriculitis requiring a combination of intraventricular gentamicin and high dose intravenous meropenem for clearance. Cerebrospinal fluid clearance occurred at 7 days after initiation of combined therapy. The patient remained shunt dependent. *Conclusions*. Meningitis and ventriculitis caused by ESBL *E. coli* species are rare and pose significant challenges to the treating physician. Early consideration for combined intraventricular and IV therapy should be made.

## 1. Introduction

Extended spectrum beta-lactamase (ESBL) producing Gram negative bacteria pose significant therapeutic challenges in the setting of infection [1–3]. The beta-lactamase producing nature of these microbes leads to resistance to commonly utilized antimicrobial agents such as penicillin and cephalosporins and intermediate resistance patterns to carbapenems [3].

In recent literature, the emergence of ESBL meningitis and ventriculitis has produced concerns as to the appropriate management strategy in these situations. Commonly reported ESBL species leading to meningitis include* Escherichia coli* [4, 5] (*E. coli*),* Klebsiella pneumoniae* [6] (*K. pneumoniae*), and* Acinetobacter* species. Controversy exists surrounding the appropriate antimicrobial regimen and the need for intraventricular or intrathecal therapy [1–6]. The need for CSF diversion also raises questions as to the appropriate medical therapy in the presence of indwelling foreign hardware.

To date, commonly utilized antimicrobial regimens for treating ESBL meningitis include high dose intravenous (IV) aminoglycoside or carbapenem based therapies, with or without intraventricular/intrathecal antimicrobials [4, 5].

Alternatively, ventriculitis secondary to ESBL species is fortunately rare and more commonly described in the pediatric population [7]. The current medical strategies in these cases are even more unclear. We describe a rare case of ESBL* E. coli* meningitis/ventriculitis in an adult and highlight the management strategy that led to successful eradication of their infection.

## 2. Case Presentations

**Figure 1:**
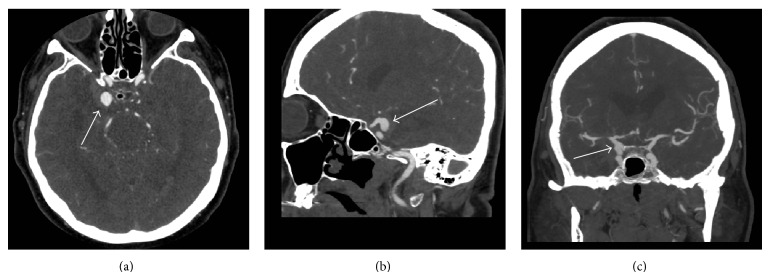
Preoperative CT-Angiogram. CT: computed tomography, CTA: computed tomographic angiography, PComm: posterior communicating artery. (a) Axial CTA displaying right PComm aneurysm (white arrow). (b) Sagittal CTA image displaying right PComm aneurysm arising from supraclinoid internal carotid artery (white arrow). (c) Coronal CTA image displaying the right PComm aneurysm (white arrow).

A 55-year-old female, with a past medical history of hypertension and smoking, was admitted to hospital for a Fisher grade 3, World Federation of Neurological Surgeons (WFNS) clinical grade 1, subarachnoid hemorrhage (SAH). Her clinical history was consistent for an acute onset severe headache and meningismus, without focal neurological deficits. The patient was afebrile on admission with a white blood cell (WBC) count of 10.1 × 10^9^/L. Computed tomographic angiography (CTA) of the Circle of Willis displayed a 1.1 cm right posterior communicating artery (PComm) aneurysm, as seen in Figure 1. Given our institutional experience with microsurgical clipping and the favorable anatomy of the aneurysm, it was elected by the treating neurosurgeon to forgo conventional angiography and arrange for microsurgical clipping. The patient was taken urgently for microsurgical clipping and placement of an external ventricular drain (EVD). The EVD was placed at 10 cm above the tragus and was open continuously in order to facilitate drainage of the thick subarachnoid blood. Cerebrospinal fluid samples were sent every second day from the drain for culture.

Postoperatively, the patient did well initially, without focal deficits. Postoperative day (POD) 1 CTA displayed obliteration of the aneurysm and no signs of ischemic insult. The patient remained EVD dependent throughout the first 8 days of her hospital course. Multiple attempts at clamping and raising the EVD led to cognitive deterioration and radiographic evidence of progressive hydrocephalus. The EVD was removed and a lumbar drain was subsequently placed, draining 10 mL per hour. On POD 11, CSF samples from the lumbar drain returned positive results for* E. coli*, with no antimicrobial sensitivities reported at that time. The patient displayed a serum WBC count of 12.5 × 10^9^/L, with a mild fever at 38.0 degrees Celsius. Urine cultures and blood cultures also returned positive for* E. coli*. Intravenous (IV) ciprofloxacin was started. On POD 12, sensitivities returned displaying a polyresistance ESBL produced* E. coli* species. The antimicrobial sensitivities displayed resistance patterns to ampicillin, trimethoprim-sulfamethoxazole, tobramycin, and ceftriaxone, with intermediate sensitivity to ceftazidime. Gentamicin and meropenem were the only two antimicrobials to which the* E. coli* species was sensitive.

Meropenem was started at 2 grams IV every 8 hours and the recommendation from infectious disease specialists was to remove the lumbar drain and avoid any foreign hardware. The lumbar drain was accidentally pulled out by the patient on POD 14, after which it was elected to monitor clinically for signs of progressive hydrocephalus. CSF samples had been still positive for* E. coli* during POD 13 and 14. On the evening of POD 14, the patient suffered a generalized tonic-clonic seizure requiring intubation and transfer to the intensive care unit. Computed tomographic (CT) imaging of the brain displayed progressive hydrocephalus; thus, an EVD was reinserted and opened at 10 cm.

**Figure 2:**
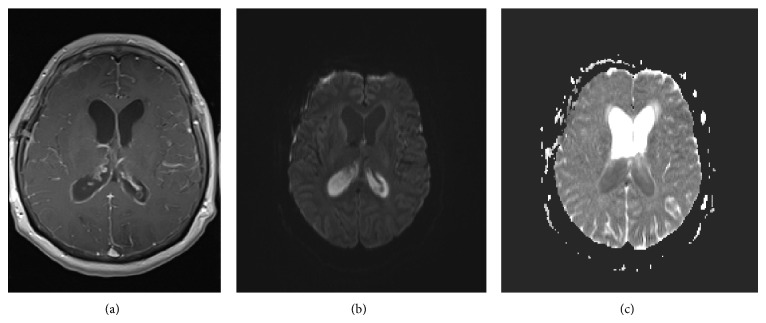
MRI brain. MRI: magnetic resonance imaging. (a) Axial T1 with gadolinium displaying ependymal enhancement in the lateral ventricles. (b) Axial diffusion weighted imaging displaying intraventricular hyperintensity, concerning restriction of ventricular sediment and consistent with ventriculitis/empyema. (c) Apparent diffusion coefficient map confirming intraventricular hypointensity and restricted diffusion of the intraventricular sediment, confirming ventriculitis/empyema.

Over the following 4 days the patient remained intubated with a depressed level of consciousness, elevated WBC count, negative electroencephalogram, and daily positive CSF samples despite ongoing IV meropenem. Magnetic resonance imaging (MRI) of the brain displayed vivid leptomeningeal and ependymal enhancement with gadolinium suggesting the ongoing presence of meningitis and ventriculitis (Figure 2). Given the MRI results, it was elected to administer combined IV meropenem and intraventricular gentamicin.

Gentamicin was administered as follows: 10 mL of CSF was removed from the EVD, 4 mg in l mL of gentamicin was injected into the EVD and flushed with 6 mL of sterile saline solution, and the EVD was then clamped for 30 min and subsequently opened. This was repeated once daily.

After 3 days of combined antimicrobial therapy, the patient's WBC count normalized and she clinically awoke, allowing for extubation. The samples of CSF first returned negative for* E. coli* after 4 days of intraventricular therapy. On POD 22, CSF samples began to return positive intermittently and so the gentamicin dosage was increased to 6 mg daily via the EVD, with IV meropenem continued at the same dose and frequency. The EVD was changed on POD 26.

With the increased dose in gentamicin, the remaining CSF samples remained negative until the patient was shunted 14 days later (POD 36). The cerebral compliance became an issue, with the patient displaying low pressure symptomatic hydrocephalus. Progressive ventricular dilation occurred with the EVD at 5 cm above the tragus. Forced drainage of 10 mL/hr via the EVD led to symptom resolution and a decrease in ventricular size. The patient had a ventriculoperitoneal shunt placed utilizing a programmable value without an antisiphon device.

## 3. Discussion

A few important points can be gleamed from our rare case of ESBL* E. coli* ventriculitis. First, the need for intraventricular/intrathecal therapy should be considered early on in the treatment course. Our case was an example of failure of high dose systemic antibiotics when utilized in isolation. Thus, combined intraventricular/intrathecal and high dose systemic therapy may need to be implemented early. Second, the dosing regimen of the intraventricular aminoglycoside had to be adjusted during the treatment course due to ongoing intermittent positive CSF samples. This highlights the need for adequate CSF dosing, of which currently there are no guidelines. Thus, constant reevaluation of the treatment regimen needs to occur. Third, persistence with combination therapy needs to occur. There is a tendency to remove all foreign hardware in the setting of meningitis/ventriculitis, due to concerns of colonization of these devices. Our case is an example where unfortunately that is not an option, due to dependence on CSF diversion. Thus, persistence with combination therapy, in addition to potentially replacing the hardware after duration of time, can lead to microbial clearance. Fourth, we waited 36 days prior to shunt placement. The reason for the delay in shunting was ongoing positive CSF samples (early on) in addition to purulent looking CSF, which we anticipated would block the shunt catheter or valve if placed too early. Thus, we delayed until the CSF was clean. Furthermore, the patient was requiring forced CSF drainage, recording negative ICP values, thus indicating an altered cerebral compliance state and low pressure hydrocephalus. Our hope was that if we waited longer, this compliance issue would begin to resolve and the chances of failing shunt placement secondary to low pressure hydrocephalus would be less. Finally, as seen in postfossa SAH, IVH, and severe meningitis cases, ventricular compliance can be altered such that the patient is left with low pressure hydrocephalus. This can pose difficult challenges for permanent shunting and valve selection.

Despite our positive results with combined IV and intraventricular antimicrobial therapy, there are both pros and cons to the administration of intraventricular/intrathecal antimicrobials. These benefits and risks need to be weighed prior to initiation of intraventricular/intrathecal antimicrobial therapy.

The benefits of intraventricular/intrathecal therapy include direct administration to the infected compartment leading to high CSF drug concentrations and potentially a higher microbe kill rate, the ability to bypass the blood brain barrier, and the potential to aid in clearing otherwise resistant infections with the central nervous system.

There are some important negative factors as to intraventricular/intrathecal antimicrobial therapy. First, given the lack of robust literature on the use of intraventricular/intrathecal antimicrobial therapy, the dosing and frequency of administration are not clear and are currently based on small series. Second, monitoring drug concentrations within the CSF can be done, but the target therapeutic levels are currently unknown. Therefore, it is difficult to know if the dose administered is adequate, outside of receiving ongoing positive CSF samples. Third, there is no consensus on the duration of therapy, and current recommendations are based on small case series and case reports. Fourth, the potential for neurotoxic side effects is concerning, especially those of ototoxicity with administration of aminoglycoside therapies. Furthermore, penicillin/cephalosporins/carbapenems cannot be administered via the intraventricular/intrathecal route given their propensity to induce seizures. Fifth, other complications described with the administration of intraventricular/intrathecal medication include aseptic meningitis, radiculitis, and meningitis. These complications can increase the risk of permanent shunt dependency. Finally, if the catheter (ventricular or cisternal) is misplaced and injections continue, this could lead to intraparenchymal injection of medications.

## 4. Conclusions

Meningitis and ventriculitis caused by ESBL* E. coli* species are rare and pose significant challenges to the treating physician. Early consideration for combined intraventricular and IV therapy should be made.
